# Entrepreneurship and career transition of elite athletes: the role of universities in supporting dual careers and successful post-sport integration

**DOI:** 10.3389/fspor.2026.1864840

**Published:** 2026-07-17

**Authors:** Bernard Massiera, Marjorie Bourse

**Affiliations:** 1 LAMHESS Laboratory, Université Côte d’Azur, Nice, France; 2 French University Sports Federation, Université Côte d’Azur, Nice, France

**Keywords:** career transition, dual career, elite athletes, entrepreneurship, university sports governance

## Abstract

**Introduction:**

The transition to a professional career following the end of an elite sporting career represents a major challenge for high-level athletes, with significant implications for their long-term well-being and sustainable professional integration. Although dual-career policies have been developed to facilitate the combination of sport and education, entrepreneurship remains a relatively underexplored pathway for post-sport career transition. This study examines the entrepreneurial dispositions of elite athletes and explores the institutional mechanisms that may facilitate their transition into entrepreneurship.

**Methods:**

A qualitative research design was adopted based on semi-structured interviews conducted with 61 elite athletes from different sporting disciplines. The interviews explored participants' perceptions of career transition, entrepreneurial intentions, perceived barriers, and support needs. The data were analysed using thematic analysis.

**Results:**

Most participants had reflected on their professional transition after sport, although only a minority had taken concrete entrepreneurial steps. Athletes identified several transferable skills acquired through elite sport, including resilience, discipline, and goal orientation, but also reported significant barriers such as limited entrepreneurial knowledge, restricted professional networks, and financial constraints. The main support needs identified included entrepreneurship training, access to professional internships, financial support, mentoring, and networking opportunities.

**Discussion:**

The findings highlight the strategic role that universities and university sports organizations can play in fostering entrepreneurial ecosystems that support athletes' dual careers and facilitate sustainable post-sport integration. Based on these findings, the study proposes a conceptual model of entrepreneurial transition designed to strengthen dual-career policies within French universities and encourage entrepreneurship as a viable post-sport career pathway.

## 1 Introduction

Elite athletes' careers are characterized by their brevity and uncertainty. Unlike traditional professional careers, sporting careers typically peak between the ages of 20 and 35, depending on the discipline, which forces athletes to consider professional transition relatively early (1). The transition to a post-sport career therefore represents a major challenge both for athletes themselves and for sports and university institutions (2).

Over recent decades, athlete career transition has attracted increasing interest in the fields of sport psychology, sport management, and public policy. Several studies have highlighted the difficulties athletes encounter when entering the labor market, particularly due to limited professional experience or interrupted academic pathways (3–5, 43). In response, many countries have developed dual-career policies designed to help athletes reconcile sporting commitments with academic or professional training (6). These policies aim to improve athletes' employability after their sporting careers while supporting athletic performance. However, most research on dual-career policies focuses primarily on academic pathways and salaried employment (7), while entrepreneurship as a transition pathway remains relatively underexplored.

Yet several studies suggest that the skills developed in elite sport—such as resilience, discipline, stress management, and performance orientation— can provide valuable assets for entrepreneurial activity (8, 9). In this context, the present research aims to analyze the entrepreneurial dispositions of elite athletes and to examine the potential role of universities in supporting these professional trajectories. More specifically, this study seeks to answer the following question: How can universities support the entrepreneurial transition of elite athletes within dual-career policies to foster successful post-sport integration?

## 2 Literature review

This section focuses on the factors determining the success of student-athletes' dual projects, which aim to reconcile academic studies with sporting careers. Achieving this balance represents a significant challenge, as combining higher education with elite sport requires strong institutional support, which remains limited in France (42, 47). Post-sport professional integration also remains difficult to implement within sports federations (10). For this reason, this section explores these characteristics and seeks to identify key determinants of successful transition.

### 2.1 Career transition of elite athletes

The career transition of elite athletes constitutes a major field of research in sport psychology, sport management, and public sport policy (11). Sporting careers are characterized by their relatively short duration and high level of uncertainty, making preparation for post-career life particularly crucial (12). Theoretical work conceptualizes athletic career development as a multidimensional process encompassing sporting, psychological, psychosocial, and academic dimensions (6). This model emphasizes that retirement from sport represents a critical phase that can generate significant adjustment difficulties, particularly when athletes have not anticipated their professional transition.

Empirical research shows that many athletes face difficulties when entering the labor market after the end of their sporting careers. These difficulties are often linked to limited professional experience, interrupted academic training, or a strong athletic identity that complicates the redefinition of professional identity (13). The literature further indicates that successful career transition depends on several factors, including social support, access to education, and early preparation for post-career life (11, 12, 44). Athletes who develop skills outside sport and maintain academic or professional engagement alongside their sporting careers are generally better prepared for this transition (2, 14).

### 2.2 Dual-career policies in sport

In response to the difficulties athletes face during career transition, sports institutions and public authorities have progressively developed dual-career policies designed to enable athletes to combine sporting careers with academic or professional training. These policies have expanded significantly in Europe over recent decades, particularly following the European Commission's adoption of guidelines supporting athletes' dual careers (15).

Dual-career arrangements rely on several institutional mechanisms, including:

- Adaptation of academic programs for athletes
- Academic tutoring systems
- Recognition of student-athlete status
- Support for professional integration

Universities play a central role in implementing these policies by offering flexible academic environments adapted to sporting constraints (16). Research shows that access to higher education contributes significantly to athletes' personal and professional development and enhances their employability after their sporting careers (17). However, implementation of dual-career policies remains uneven across countries and institutions, and athletes continue to face difficulties in balancing academic and sporting commitments (18).

Moreover, most dual-career initiatives still focus primarily on higher education access and salaried employment outcomes (19). Entrepreneurship as a transition pathway remains largely absent, even though elite athletes develop competencies directly relevant to entrepreneurial activity. The following section therefore examines entrepreneurship as an alternative pathway that remains underexplored but particularly relevant to athletes' career transitions.

### 2.3 Entrepreneurship as a pathway for professional transition

Entrepreneurship represents a potentially well-suited transition pathway for elite athletes. Research in entrepreneurship suggests that characteristics associated with entrepreneurial success—such as perseverance, risk tolerance, and performance orientation—are also prevalent among elite athletes (20, 21). Skills developed through high-level sport can therefore be considered transferable competencies conducive to entrepreneurial activity.

Elite athletes possess strong entrepreneurial potential due to their capacity to operate in competitive and uncertain environments (22). Furthermore, their media visibility and social networks may represent valuable resources for entrepreneurial ventures. Nevertheless, several studies indicate that athletes face substantial obstacles when attempting to create businesses, including limited management knowledge, difficulties accessing financing, and professional networks largely confined to the sporting environment (21, 23, 24). These findings highlight the importance of entrepreneurial support mechanisms, such as training programs, mentoring networks, and business incubators, within which universities may play a central coordinating role.

### 2.4 The role of universities in the entrepreneurial ecosystem

Universities occupy a strategic position in the development of entrepreneurial ecosystems. Over recent decades, many universities have established programs aimed at encouraging student entrepreneurship, including university-based incubators and entrepreneurship training programs. In the field of sport, universities can play a particularly important role in supporting athletes' entrepreneurial trajectories due to their position at the intersection of educational and sporting systems. Research on sport governance highlights universities as key actors within national sport systems, contributing both to talent development and elite athlete education (25). In some countries, particularly the United States, university sport plays a central role in athlete development and preparation for professional careers within sport or other economic sectors. University programs can therefore provide environments conducive to the development of entrepreneurial skills. However, in many European countries, relationships between universities and national sport systems remain less integrated, limiting universities' potential role in athlete entrepreneurship support. In this context, entrepreneurship programs specifically designed for athletes represent an innovative institutional approach capable of strengthening integration between educational and sporting systems.

## 3 Methods

This study adopts an exploratory qualitative approach aimed at examining elite athletes' perceptions, experiences, and needs regarding their transition into entrepreneurship (26). Given the complexity and relatively underexplored nature of this phenomenon, a qualitative methodology is particularly well suited to understanding individual trajectories and complex transition processes (26). This research is situated within a body of work that focuses on exploring athletes' lived experiences and perceptions (27). To achieve this, we adopted an interpretive approach, viewing athletes' perceptions as socially constructed and shaped through interactions within their specific social and cultural contexts (28).

### 3.1 Methodological approach

A qualitative research design was selected to enable an in-depth understanding of athletes' subjective experiences and the dynamics associated with their career transitions. This approach is particularly relevant for investigating contextual, multidimensional, and difficult-to-quantify phenomena (29). Several measures were implemented to enhance the rigor, credibility, and transparency of the study (30). These included a clearly defined sampling strategy, explicit participant selection criteria, systematic data collection procedures, and a structured analytical framework (31). The analysis was conducted manually, without the use of specialized qualitative data analysis software, to maintain a close, iterative, and reflexive engagement with the data throughout the coding and theme development process. Data were collected through semi-structured interviews, allowing for both consistency across the themes explored and sufficient flexibility to investigate individual experiences in greater depth. This method is particularly well suited for examining career pathways and professional transitions within the sporting context.

### 3.2 Participant selection

Participants were recruited through purposive sampling, a method commonly used in qualitative research to select individuals with experience relevant to the phenomenon under investigation. Recruitment was conducted through university networks, sports federations, professional sport networks, and online platforms.

The inclusion criteria were as follows:

- being active in a sporting career;
- be active in a sports career or have retired from competition within the last two years;
- having considered a future career transition;
- expressing an interest in entrepreneurship.

The final sample consisted of 60 participants, providing diversity in terms of sporting disciplines, competitive levels, and educational backgrounds (Table 1). This diversity enhanced both the analytical richness and the transferability of the findings.

**Table 1:** Characteristics of the 60 participants interviewed.

Level	53 elite athletes	6 professionals	1 in training
Age	18–20 years (62%)	21–23 years (26%)	24 and over (12%)
Gender	Men (56%)	Women (44%)	
Disciplines	Team sports (54%)	Individual (46%)	
Practices	Variety of activities (football, rugby, swimming, athletics, cycling)		
Training in entrepreneurship	46 are seeking training in entrepreneurship,		
	41 plan to develop a professional network,		
	33 wish to receive support in finding funding,		
	27 would like to participate in workshops or seminars.		

### 3.3 Interview procedure

The interviews were conducted between November 2024 and March 2025, either in person or remotely via videoconferencing platforms. Their duration ranged from 30 to 60 min. Prior to each interview, participants were informed about the objectives of the study and provided their informed consent. With participants' consent, the interviews were audio-recorded and transcribed verbatim to ensure the accuracy and reliability of the data, in line with established qualitative research practices (26, 32).

### 3.4 Interview guide

This semi-structured interview guide was developed through a rigorous and iterative process. First, it was informed by the existing literature on athlete career transition, dual-career policies, and sport entrepreneurship to ensure a strong theoretical foundation (26, 33). The questions were designed to reflect the objectives of the study and to explore the main dimensions of athletes' experiences and perceptions. To enhance the reliability and validity of the data collection instrument, the interview guide was progressively refined through collaborative discussions between the researchers, aimed at improving its internal coherence and content validity. Prior to the main phase of data collection, the guide was pilot tested with a small group of athletes to assess the clarity, relevance, and comprehensibility of the proposed questions. Feedback gathered during this pilot phase led to several minor revisions to the wording and sequencing of the questions. Following this iterative process, a final version of the interview guide was formalized and adopted for data collection (Table 2).

**Table 2:** Interview guide structure.

(1). Athletic Career Path – Could you describe your athletic career path? – At what level did you compete? – How would you describe your experience as an elite athlete? (2). Preparation for Post-Sport Career Transition – Have you thought about your career after sport? – When did you begin considering this transition? – What are your main concerns regarding life after sport? (3). Perceptions of Entrepreneurship – Have you ever considered starting your own business? – What aspects of entrepreneurship appeal to you? – Which skills acquired through sport do you believe could be useful for entrepreneurship? (4). Barriers to Entrepreneurship – In your opinion, what are the main obstacles to starting a business? – Do you believe you possess the skills required to become an entrepreneur? – What resources do you currently lack? (5). Support Needs – What type of support would be most beneficial to you? – What role could universities play in supporting athletes’ entrepreneurial development? – What initiatives or support mechanisms could facilitate athletes’ entrepreneurial projects?

### 3.5 Data analysis

The analysis was conducted through several successive stages (Table 3). The data collected from the athletes were analysed using thematic analysis (34) in line with recent methodological developments in sports science (27). To enhance the reliability of the analysis, coding was performed independently by both researchers, and any disagreements were resolved through discussion until a consensus was reached. An audit trail documenting coding decisions and theme development was maintained throughout the analysis process. Saturation was reached around interview 45, when no new themes emerged and was confirmed through subsequent interviews.

**Table 3:** Phases of interview analysis.

(1) Transcription → (2) Coding → (3) Theme Refinement → (4) Theme Evaluation → (5) Interpretation

#### 3.5.1 Coding process framework

A systematic coding process was employed to identify, organize, and interpret the themes emerging from the data (Table 4). First, the interview data were transcribed, enabling the identification of initial ideas and patterns. Second, an inductive coding process was carried out by identifying meaningful units within the participants' narratives. The coding was conducted independently by both researchers. This process continued until thematic saturation was reached, defined as the point at which no new themes emerged from the data. In the third stage, themes relevant to the research objectives were refined through a systematic comparison with the collected data. The coherence and consistency of the themes were then assessed through collaborative discussions between the two researchers, with disagreements resolved through consensus. Finally, the themes were formalized in the Discussion section, linked to the relevant scientific literature, and partially illustrated through selected interview excerpts chosen for their explanatory value.

**Table 4:** Codebook for thematic analysis.

Code	Definition	Inclusion criteria	Exclusion criteria	Example quote
Theme 1: Anticipation of career transition				
Awareness of career end	Recognition of limited duration of career	Mentions inevitability of retirement	General career concerns	A sports career doesn't last a lifetime.
Early career planning	Reflection before retirement	Planning during athletic career	After retirement only	It's important to think about it early.
Passive anticipation	Awareness without action	Concern without action	Active preparation	We think about it, but don't act.
Time constraints	Limited time due to sport	Training/travel constraints	Financial barriers	It's hard to focus on anything else.
Theme 2: Transferable skills				
Discipline	Structured effort	Training habits	General motivation	We do everything to achieve goals.
Resilience	Coping with setbacks	Recovery from failure	Stress only	We never give up.
Stress management	Handling pressure	Competition stress	Emotional issues	We're used to pressure.
Goal orientation	Setting objectives	Strategic planning	General ambition	We set clear goals.
Adaptability	Adjusting to uncertainty	Changing environments	Routine	We constantly adapt.
Theme 3: Barriers to entrepreneurship				
Lack of technical skills	Business knowledge deficit	Finance/management gap	Soft skills	We lack technical knowledge.
Limited network	Sports-only network	Lack external contacts	Large network	Our network is mostly in sports.
Financial risk	Income uncertainty	Investment fear	General fear	Starting a business is risky.
Identity barriers	Difficulty leaving athlete role	Strong athletic identity	General doubt	Hard to imagine another role.
Structural constraints	System limitations	Lack resources/programs	Individual limits	We lack access to resources.
Theme 4: Institutional support needs				
Entrepreneurship training	Need for formal education	Courses/workshops	Informal learning	We need training.
Mentorship	Need for guidance	Mentors/coaches	Peers only	Guide would help.
Networking opportunities	Expand contacts	Events outside sport	Personal network	We need to meet people.
Financial support	Need funding	Grants/investors	General economy	Funding is essential.
Role of universities	Expectations of HEIs	University programs	Other institutions	Universities could support us.

The codebook was developed inductively following established thematic analysis procedures, including iterative coding, category refinement, and the definition of inclusion and exclusion criteria (34).

Following this step, a participant verification process was conducted to strengthen the credibility of the analysis. This process was carried out manually, through direct communication with participants. Some participants were contacted following the initial analysis phase and received summaries of the results. They were asked to provide feedback on the accuracy, clarity, and representativeness of the interpretations. Minor clarifications were made where necessary. This process ensured that the results remained fully aligned with the participants' perspectives and lived experiences.

## 4 Results

The thematic analysis of the results identified four main themes structuring athletes' representations and experiences: (1) anticipation of career transition, (2) transferable skills, (3) barriers to entrepreneurship, and (4) needs for institutional support (Table 5). Although the themes were inductively derived from the data, they are consistent with existing literature on athlete career transitions and sport entrepreneurship, particularly regarding the importance of career anticipation (12), transferable skills (22), structural barriers (23), and the need for institutional support (18).

**Table 5:** Summary of themes and subthemes from thematic analysis.

Theme	Subthemes
Anticipation of career transition	Awareness of career end; Early reflection; Limited action; Time constraints
Transferable skills	Discipline; Perseverance; Stress management; Goal orientation; Adaptability
Barriers to entrepreneurship	Lack of technical skills; Limited network; Financial risk
Institutional support needs	Entrepreneurship training; Mentoring; Networking opportunities; University support programs

Themes and subthemes were inductively derived from the data following thematic analysis (34).

### 4.1 Anticipation of professional transition

The interviews reveal that most of the athletes surveyed are aware of the need to prepare for their career transition. The end of a sports career is generally seen as an inevitable step, often anticipated due to physical limitations and the uncertainty inherent in elite sports. Most participants (approximately 65%–70%) mention the relatively short duration of a sports career, which prompts them to start thinking about their professional future as early as the midpoint of their career. One participant notes: “A sports career doesn't last a lifetime. We know that at some point, we'll have to do something else, so it's important to think about it early on.” (Athlete 01). However, the interviews also show that this awareness does not always translate into concrete action. Approximately 80% of participants reported having reflected on their career transition after sport, whereas fewer than 20% indicated that they had undertaken concrete entrepreneurial actions. The demands of training and competition often limit athletes’ ability to invest in parallel professional projects. Another athlete notes: “Between training, competitions, and travel, it's hard to focus on anything else. We think about it, but we don't always have the time to act.” (Athlete 04). These findings highlight a disconnect between the awareness of the need to prepare for life after sports and the actual implementation of career transition strategies.

This anticipatory awareness is also reflected in athletes' narratives. Some participants highlight a growing need to project themselves beyond sport: “At some point, you realise that performance won't last forever, so you need to start thinking about what comes next” (Athlete 03). Others emphasize the difficulty of translating this awareness into action, noting that “during your career, you focus on sport first, and the rest often comes later” (Athlete 04).

### 4.2 Transferable skills developed through sport

The interviews reveal that athletes identify several skills developed during their athletic careers that could be useful in an entrepreneurial context. A large majority of participants (approximately 70%–90%) identified discipline, perseverance, stress management, a competitive mindset, and teamwork as their most prominent skills. These skills are often seen as important assets for managing an entrepreneurial project. One participant comments: “High-level sports teach us never to give up. When you have a goal, you do everything to achieve it. I think that's an important quality for entrepreneurship.” (athlete 02). Similarly, some athletes highlight their ability to thrive in uncertain and competitive environments, which they believe is an advantage in the entrepreneurial world. Another participant explains: “In sports, we're used to pressure and uncertainty. We must constantly adapt and find solutions. I think that can be useful for starting a business.” (athlete 03). However, several participants also acknowledge that these skills must be complemented by technical knowledge in management and entrepreneurship. Approximately 50%–70% of participants emphasized strategic skills that are strongly developed in elite sport contexts yet remain comparatively underexplored in the entrepreneurship literature. These include the ability to set clear goals, structure an action plan, and maintain a high level of discipline to achieve them. Athletes also highlight their ability to bounce back after a setback, an essential quality when facing the uncertainty and unpredictability inherent in the early stages of starting a business. These skills—perseverance, managing adversity, and goal orientation—appear to be genuine assets that can be transferred to entrepreneurial activity.

### 4.3 Obstacles to entrepreneurship

Despite their interest in entrepreneurship, athletes identify several obstacles that can hinder the development of entrepreneurial projects. The main obstacle mentioned is a lack of knowledge in management and entrepreneurship. Several participants explain that they lack the necessary skills to start and run a business. One participant laments: “We have the interpersonal skills, but we lack the technical knowledge. We don't necessarily know how to start a business” (Athlete 09). The lack of a professional network is also a significant obstacle. Athletes explain that their social network is often composed mainly of other athletes, which can limit access to entrepreneurial resources. One participant notes: “Our network is mostly in sports. To start a business, you need to know people in other fields.” Finally, financial uncertainty is also perceived as a major barrier to entrepreneurship. An athlete explains: “Starting a business always involves financial risk. When you leave sports, you often seek a certain stability” (Athlete 11). These results highlight the need for support mechanisms capable of reducing these barriers.

These competencies are widely recognized by the athletes themselves. Some participants emphasize the importance of discipline and long-term goal setting: “High-level sport teaches us rigor and the ability to set clear objectives over time” (Athlete 05). Others highlight their capacity to cope with uncertainty and failure: “We learn how to bounce back after setbacks, which is essential when starting a project” (Athlete 06). In addition, several respondents refer to practical and operational skills developed through sport, such as decision-making and teamwork: “On the field, as in a project, you have to be reactive and able to make quick decisions” (Athlete 14). These testimonies reinforce the idea that elite sport fosters a set of transferable competencies that can be mobilized in entrepreneurial contexts, particularly in environments characterized by uncertainty, pressure, and the need for continuous adaptation.

### 4.4 Institutional support needs

The interviews reveal that 90% of athletes express a strong need for support in developing their career plans. Participants mention several types of support programs. First, athletes emphasize the importance of access to entrepreneurship training that allows them to acquire skills in management, marketing, and finance. One participant explains: “If we had access to training tailored to our schedule, that would be really helpful” (Athlete 13). Athletes also highlight the importance of mentoring and personalized support. One participant note: “Having someone who understands the business world guide us could really help us structure our projects” (Athlete 14) Finally, participants emphasize the potential role of universities in establishing entrepreneurial support programs. Several athletes suggest that universities could develop specific programs tailored to athletes, such as incubators or entrepreneurship programs. One participant state: “Universities could really play an important role in helping us develop entrepreneurial projects” (Athlete 21). This need for support is also reflected in athletes' expectations regarding concrete and accessible programs: “We need support that fits our training schedule, otherwise it's impossible to get involved” (Athlete 25). Others also emphasize the importance of networking opportunities beyond the sports environment: “Meeting people outside of sport would really help us develop our projects” (Athlete 20). These results suggest that universities could be key players in developing an entrepreneurial ecosystem tailored to athletes.

## 5 Discussion

This section discusses the main findings of the study by highlighting the key factors shaping athletes' engagement in entrepreneurship. It examines both individual dimensions—such as skills and identity—and broader structural constraints within the French dual-career system, to provide a comprehensive understanding of the opportunities and challenges faced by student-athletes in their entrepreneurial transition.

### 5.1 Athletes' entrepreneurial skills: strengths and limitations

About entrepreneurial competencies, our findings confirm the literature showing that athletes possess a range of behavioural and psychosocial skills that are conducive to entrepreneurship. Some athletes develop characteristics commonly associated with entrepreneurs, such as risk tolerance, resilience, and the ability to operate in uncertain environments (20).

Similarly, research in sport entrepreneurship tends to portray athletes as individuals with a strong entrepreneurial orientation due to their experience in highly competitive and performance-driven contexts (20, 21). However, our findings provide a more nuanced perspective on this body of work. While athletes acknowledge the transferability of their skills, they also emphasize a lack of technical knowledge in areas such as management, finance, and marketing. They further identify the demands associated with pursuing a dual career as a limiting factor in their entrepreneurial preparation. In this respect, our results are consistent with studies showing that high levels of psychological capital among athletes do not necessarily translate into sufficient entrepreneurial capital to successfully launch a business, due to deficiencies in technical skills, time constraints, and structural barriers (23, 35).

### 5.2 Athletic identity and its ambivalent role in entrepreneurial transitions

These findings can be further interpreted considering recent research on athletic identity, as well as contemporary approaches to professional identity and career transitions. The literature suggests that a strong athletic identity acts both as a resource and as a source of resistance during career transition processes (17, 48). On the one hand, athletic identity fosters the development of key competencies such as perseverance, commitment, discipline, and a performance-oriented mindset. It also contributes to the development of substantial psychological capital, facilitating adaptation to uncertain and competitive environments. On the other hand, an excessively exclusive identification with the athlete role may lead to identity foreclosure, characterized by a premature and narrowly focused commitment to a single role (36). In the contemporary sporting context, several studies have shown that elite athletes tend to develop a highly unidimensional identity centred on their sporting careers, which may hinder their ability to envision alternative professional trajectories (37, 49).

Our findings reveal a form of identity-based resistance toward life after sport. This resistance is reflected in difficulties imagining oneself in alternative professional roles, particularly entrepreneurial ones, which are often perceived as distant from the norms and values of the sporting environment. Recent research in sport psychology suggests that this difficulty is reinforced by the intensity of athletes' identity investment in sport, as well as by the early structuring of life trajectories around performance objectives (50). From the perspective of social identity theory, this resistance can be interpreted as an attempt to maintain continuity with one's reference group, which may limit openness to other social and professional environments (38). Furthermore, athletic retirement often involves a loss of status, recognition, and structured routines, generating identity disruption and heightened uncertainty (12).

This dynamic confirms that career transition is not merely a professional adjustment but rather a process of identity reconstruction. Recent studies emphasize the importance of developing a pluralistic and evolving identity that enables athletes to mobilize their skills across different contexts (39). From this perspective, while entrepreneurship appears to represent a promising post-sport career pathway for athletes, its successful realization requires appropriate support incorporating a strong identity-development dimension. Such support should not only facilitate the transformation of athletic competencies into operational entrepreneurial skills but also foster an active and athlete-specific process of professional transition.

### 5.3 Structural barriers to entrepreneurship within the French dual-career system

Entrepreneurship remains underdeveloped within French dual-career programs due to several structural factors. First, these programs have historically been oriented toward integration into salaried employment, particularly within the public sector or sport-related professions. This orientation can be partly explained by the desire of institutions to secure athletes' professional futures by directing them toward career paths perceived as more stable and predictable.

These findings are consistent with existing research on dual-career systems in Europe, which shows that public policies largely prioritize academic success and entry into the salaried labor market, often at the expense of alternative career pathways such as entrepreneurship (18, 19). In this respect, our results confirm that institutional frameworks remain strongly oriented toward traditional employability outcomes rather than toward the development of entrepreneurial trajectories.

In France, for example, many elite athletes benefit from tailored career arrangements within public institutions—including the police, military, and local authorities—which enable them to combine sport and employment. While these mechanisms provide important stability, they may also limit the exploration of more uncertain pathways such as entrepreneurship. This observation corroborates previous research indicating that athletes are frequently guided toward predefined and secure professional trajectories rather than encouraged to explore alternative career options (7, 10). Within the university setting, this logic is reflected in the strong emphasis placed on academic qualifications, which are primarily viewed as pathways to salaried employment. Dual-career support systems therefore focus on academic achievement and the acquisition of transferable qualifications aligned with established professions, rather than on business creation. In line with the literature, entrepreneurship remains marginal within these programs, often perceived as a riskier and less institutionally supported route (18).

Consequently, athletes have limited exposure to entrepreneurial practices and environments. Our findings highlight that it is uncommon for dual-career programs to include entrepreneurship training, incubator experiences, or structured links with the business world. This result is consistent with prior studies pointing to fragmented support systems and insufficient coordination between educational, sporting, and economic actors (2, 18). A key implication of this structural configuration is the persistence of a gap between the competencies developed through sport and their application to entrepreneurial activities. As shown in previous research, this lack of integration contributes to a deficit in technical and managerial skills as well as limited access to professional networks outside the sporting environment (21, 23). Taken together, these findings suggest that structural constraints—rather than solely individual limitations—play a central role in shaping athletes’ entrepreneurial engagement. Our study therefore extends the literature by highlighting how the institutional configuration of the French dual-career system may restrict the emergence of entrepreneurial pathways, despite athletes' underlying potential and interest in this domain.

## 6 Public policy recommendations

This section presents a set of recommendations aimed at supporting the career transition of elite athletes, with a particular focus on entrepreneurship. Drawing on both international best practices and the findings of this study, it highlights the potential role of universities and institutional partnerships in developing more integrated and effective dual-career pathways.

### 6.1 France considering international experience

At the European level, this trend is supported by the literature, which shows that public policies on dual careers largely prioritize traditional employability and integration into the salaried labour market (18). Within this framework, entrepreneurship often remains marginalized, being perceived as a riskier option and less compatible with the objective of ensuring secure professional career paths (40).

In contrast, the American model stands out for its more diversified approach and greater openness to entrepreneurial career trajectories. The university system, structured around the NCAA, promotes the early integration of professional development issues into the student-athlete experience (45, 46). American universities offer not only academic education, but also specific initiatives designed to foster entrepreneurial skills, including incubation programs, entrepreneurship courses within business schools, start-up competitions, and facilitated access to innovation ecosystems. For example, some universities enable student-athletes to participate in incubator programs or benefit from mentorship provided by entrepreneurs, thereby encouraging the development and testing of business ideas during their university years.

Furthermore, the American economic and institutional culture places greater value on individual initiative and risk-taking, making entrepreneurship a more legitimate post-sport career option. Programs such as NCAA Life Skills and initiatives developed by the United States Olympic and Paralympic Committee (USOPC) (41) explicitly support a wide range of professional pathways, including entrepreneurship, by providing tailored training, networking opportunities, and career development resources. This comparison highlights a major structural difference: while the French model prioritizes stability and continuity through salaried employment, the American model places greater emphasis on career diversification and the exploration of entrepreneurial opportunities. As a result, French athletes generally have fewer institutional opportunities to develop entrepreneurial projects, limiting their ability to leverage their skills and experiences in innovative and business-oriented environments.

### 6.2 The role of French universities in dual-career pathways

The findings of this study highlight the crucial role that French universities can play in supporting the career transitions of elite athletes. Historically committed to facilitating dual-career pathways, universities have progressively developed mechanisms such as academic flexibility, tutoring, and individualized support services. However, the interviews reveal that while universities provide a favourable environment for personal development and employability, their initiatives remain primarily focused on academic achievement and entry into salaried employment.

The findings also indicate that many athletes express a strong interest in entrepreneurship, which they perceive as a viable post-sport career pathway that allows them to leverage their unique competencies—including discipline, adversity management, goal setting, and resilience—as well as their networks and sporting identity.

In this context, the study's conclusions point to the need for a clearer framework defining the role of universities in supporting the entrepreneurial transition of student-athletes. Universities could strengthen the integration of entrepreneurship education within existing support systems for elite athletes by offering flexible training formats—such as workshops, short courses, and certification programs—focused on business creation, project management, and opportunity recognition, while considering the specific constraints associated with high-level sport participation.

### 6.3 Modelling the evolution of the French system

**Figure 1:**
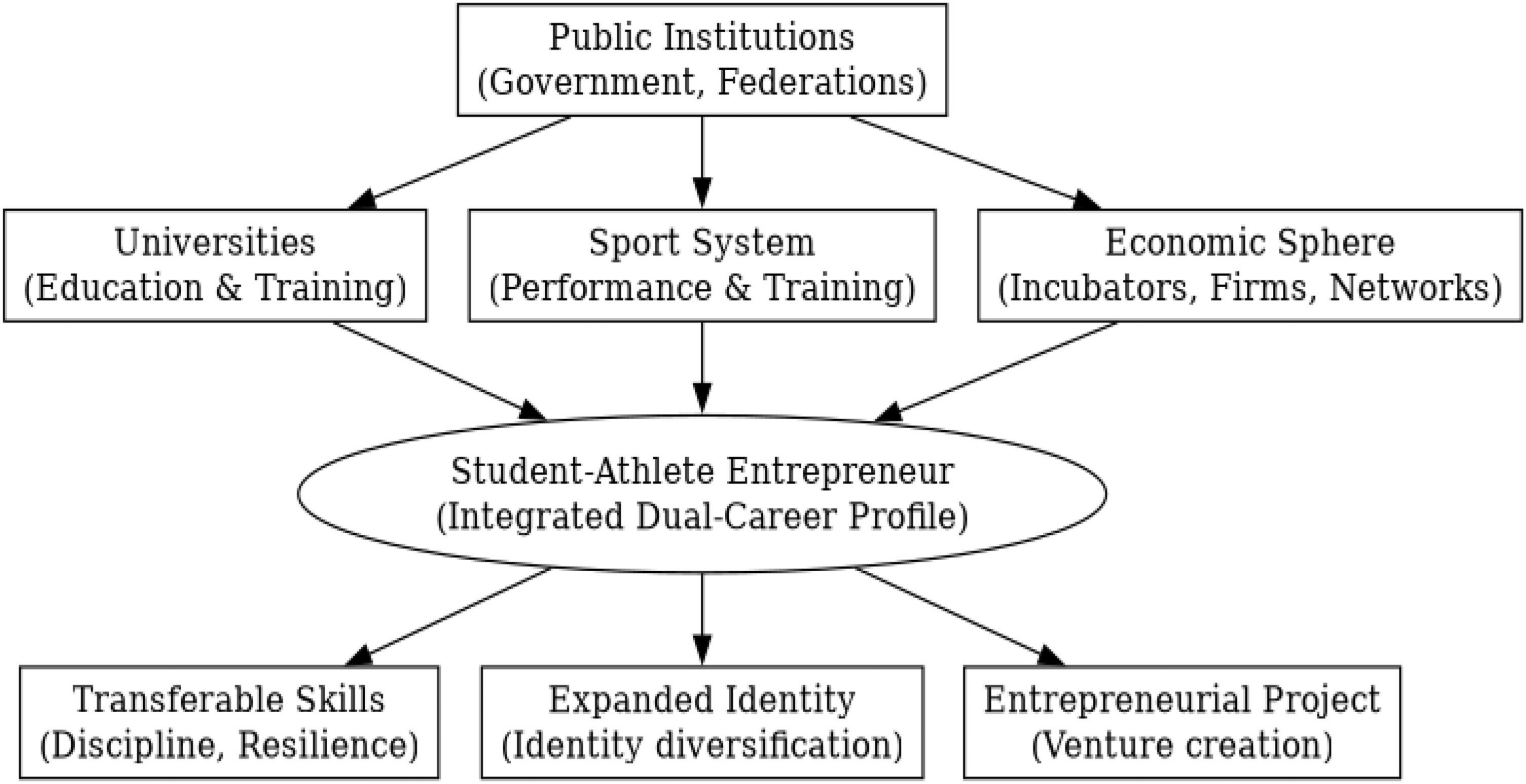
Integrated dual-career entrepreneurial system for student-athletes. Diagram developed by the authors based on the U.S. model for supporting athletes nearing the end of their careers in their transition to entrepreneurship.

Drawing inspiration from the American model, it is possible to envision the French system evolving toward an integrated dual-career framework that coherently combines the academic (universities), athletic (sports federations, INSEP, and elite training centers), and economic and entrepreneurial (incubators, companies, and professional networks) dimensions. Unlike the current framework, which is primarily focused on salaried employment, this system aims to diversify post-athletic career paths (Figure 1). This model fully integrates entrepreneurship as a career option, structured around five key pillars.

#### 6.3.1 Pillar 1: institutional integration

Drawing inspiration from the NCAA model, the proposed system should rely on strong coordination between higher education institutions and sports organizations.

**Objective:** Reduce the current fragmentation of support mechanisms and establish a clear, coherent, and structured pathway for student-athletes interested in entrepreneurship.

Key actions include:

- Establishing an enhanced *student-athlete entrepreneur* status that formally recognizes dual sporting and entrepreneurial commitments.
- Strengthening collaboration among key stakeholders (Universities, Sports federations, Ministry of Sports, Entrepreneurial support structures such as incubators and accelerators).

Such institutional integration would facilitate access to resources, improve information sharing, and create a more supportive environment for entrepreneurial development among athletes.

#### 6.3.2 Pillar 2: integrated entrepreneurial education

**Objective:** Address the entrepreneurial skills gap identified among athletes by providing structured access to entrepreneurship education and interdisciplinary training opportunities.

Universities should integrate entrepreneurship modules covering topics such as:

- Venture creation and business development;
- Business model design;
- Sports marketing and brand management;
- Entrepreneurial finance and fundraising.

Given athletes' specific time constraints, educational formats should be adapted to their sporting commitments through:

- Short and intensive workshops;
- Professional certifications (e.g., a University Diploma in Sports Entrepreneurship);
- Flexible e-learning and hybrid learning programs.

These initiatives would enhance athletes’ entrepreneurial competencies while maintaining compatibility with high-performance sport participation.

#### 6.3.3 Pillar 3: dedicated incubation structures for athletes

**Objective:** Transform entrepreneurial intentions into viable business projects through specialized incubation programs and business-oriented entrepreneurial ecosystems.

To achieve this objective, hybrid sport–university incubators should be developed, fostering collaboration between the academic, sporting, and entrepreneurial spheres.

Strategic partnerships could involve:

- Station F and regional incubators;
- Sports federations;
- Corporate partners and sponsors.

Support services should include:

- Mentoring from former athlete-entrepreneurs;
- Access to investors and funding opportunities;
- Individualized project coaching and business development support.

Such structures would provide athletes with the practical guidance and resources necessary to successfully launch and sustain entrepreneurial ventures.

#### 6.3.4 Pillar 4: identity development and career transition

**Objective:** Reduce identity-related barriers to entrepreneurship by supporting athletes in the development of entrepreneurial self-concepts and facilitating the transition from a sport-centered identity to a broader professional identity.

Recommended initiatives include:

- Identity and career coaching programs;
- Career planning and projection workshops;

Training focused on:

- The development of multiple professional identities;
- The transferability of athletic competencies to entrepreneurial contexts;
- Career transition and retirement management.

By fostering identity diversification, these initiatives can strengthen entrepreneurial engagement and improve long-term career adaptability.

#### 6.3.5 Pillar 5: networks and social capital development

**Objective:** Expand athletes' access to entrepreneurial opportunities by helping them move beyond exclusively sport-based networks and build valuable social capital within the business ecosystem.

This pillar should promote the development of networks involving:

- Former athlete-entrepreneurs;
- Business professionals and industry experts;
- Entrepreneurial mentors and investors.

Relevant initiatives include:

- Networking events;
- Investor–athlete matchmaking sessions;
- Entrepreneurship competitions and sport-focused hackathons.

Strengthening social capital would facilitate knowledge exchange, opportunity recognition, resource acquisition, and ultimately increase the likelihood of entrepreneurial success among athletes.

By fully integrating entrepreneurship into their dual-career policies, French universities could position themselves as key players in athletes' post-career transitions, complementing the role of traditional sports organizations.

## 7 Conclusion

This study highlights the relevance of entrepreneurship as a promising yet underdeveloped pathway for the career transition of elite athletes. While athletes possess strong behavioural and psychosocial competencies—such as resilience, discipline, and the ability to perform under pressure—these assets alone are not sufficient to ensure successful entrepreneurial ventures. The findings reveal a clear gap between athletes' entrepreneurial potential and their access to the technical knowledge, networks, and institutional support required to bring projects to fruition.

The results also emphasize the ambivalent role of athletic identity, which can both facilitate and hinder career diversification. This underscores the importance of developing a more holistic approach to athlete support, one that encourages the construction of multifaceted identities and transferable skill sets beyond sport. In this context, dual-career policies must evolve to integrate entrepreneurship more explicitly as a viable career option.

Universities emerge as key actors in this transformation. Positioned at the intersection of education, sport, and professional development, they are uniquely equipped to foster entrepreneurial ecosystems tailored to athletes' specific needs. By embedding entrepreneurship education, strengthening partnerships with external stakeholders, and providing flexible, targeted support mechanisms, universities can play a decisive role in bridging the gap between sporting careers and sustainable professional futures.

Ultimately, supporting athletes' entrepreneurial transition requires a coordinated effort involving academic institutions, sports organizations, and public actors. Future research could further explore the long-term impact of such initiatives and compare different international models. Advancing this agenda will contribute not only to improving athletes’ post-career outcomes but also to enriching the broader entrepreneurial landscape.

## Data Availability

The original contributions presented in the study are included in the article/Supplementary Material, further inquiries can be directed to the corresponding author/s.

## References

[1] Tanaka H Toussaint J-F. Editorial: growth, peaking, and aging of competitive athletes. Front Physiol. (2023) 14:1165223. 10.3389/fphys.2023.1165223 36909222 PMC9992958

[2] Di Rocco F Romagnoli C Ciaccioni S Capranica L Padua E Guidotti F. Sustainable career transitions and mental health support in elite sport: a systematic review of evidence and practices. Sports. (2025) 13(12):438. 10.3390/sports13120438 41441422 PMC12813639

[3] Robnik P Kolar E Štrumbelj B Ferjan M. Dual career development perspective: factors affecting quality of post-sport career transition of employed Olympic athletes. Front Psychol. (2022) 12:800031. 10.3389/fpsyg.2021.800031 35058859 PMC8764152

[4] Schmid J Conzelmann A Engel RR Kuettel A Schmid MJ. Retirement from elite sport and self-esteem: a longitudinal study over 12 years. Front Psychol. (2023) 14:1176573. 10.3389/fpsyg.2023.1176573 37213388 PMC10197962

[5] Sides R Osborn DS Walker I Stamm J Villarreal B. Beyond the game: exploring the interplay of career thoughts, career adaptability, and athletic identity in shaping postgraduation paths for student athletes. J Postsc Stud Success. (2025) 4(4):89–116. 10.33009/fsop_jpss135618

[6] Wylleman P Alfermann D Lavallee D. A developmental perspective on transitions faced by athletes. Psychol Sport Exerc. (2004) 5(1):7–20. 10.1016/S1469-0292(02)00049-3

[7] Nyberg C Wagnsson S Gustafsson H Stråhlman O. Dual career support among world-class athletes in Sweden: performance, education, and employment. Front Psychol. (2023) 13:1093562. 10.3389/fpsyg.2022.1093562 36687839 PMC9846237

[8] Gupta S McCarthy PJ. The sporting resilience model: a systematic review of resilience in sport performers. Front Psychol. (2022) 13:1003053. 10.3389/fpsyg.2022.1003053 36619099 PMC9811683

[9] Haski S Moustakas L Heinzen M von Korflesch H Kalina L Stanescu R. From athlete to entrepreneur? Investigating the influence of sport characteristics on athlete’s entrepreneurial orientation competencies. Manag Sport Leis. (2024) 30:826–44. 10.1080/23750472.2023.2299830

[10] Hong HJ Minikin B. An international analysis of career assistance programmes for high-performance athletes. Int J Sport Policy Politics. (2023) 15(4):705–24. 10.1080/19406940.2023.2242873

[11] Monteiro R Monteiro D Torregrossa M Travassos B. Modeling athletic career of football players: implications for career management and retirement. Int J Sports Sci Coach. (2022) 18:1478–86. 10.1177/17479541221111616

[12] Park S Lavallee D Tod D. Athletes’ career transition out of sport: a systematic review. Int Rev Sport Exerc Psychol. (2013) 6(1):22–53. 10.1080/1750984X.2012.687053

[13] Alfermann D Stambulova N. Career transitions and career termination. Psychol Sport Exerc. (2007) 8(1):1–3. 10.1016/j.psychsport.2006.03.003

[14] De Maio M Montenegro S Papale O Serafini S Prestanti I Izzicupo P Abalasei B-A. A scoping review of student-athletes’ perspectives on dual career policies, provisions and challenges. Front Sports Act Living. (2025) 7:1566208. 10.3389/fspor.2025.1566208 40529403 PMC12170630

[15] European Commission. EU guidelines on Dual Careers of Athletes. Luxembourg: Publications Office of the European Union (2012).

[16] Aquilina D. A study of the relationship between elite athletes’ educational development and sporting performance. Int J Hist Sport. (2013) 30(4):374–92. 10.1080/09523367.2013.765723

[17] Stambulova NB Ryba TV Henriksen K. Career development and transitions of athletes: the international society of sport psychology position stand revisited. Int J Sport Exerc Psychol. (2021) 19(4):524–50. 10.1080/1612197X.2020.1737836

[18] Guidotti F Cortis C Capranica L. Dual career of European student-athletes: a systematic literature review. Kinesiol Sloven. (2015) 21(3):5–20.

[19] Varmus M Mičiak M Toman D Jastraban M Kuljovský M Sobol J. Athletes’ education for their successful future career after sports—perspective of former athletes and potential employers. Adm Sci. (2025) 15(2):46. 10.3390/admsci15020046

[20] Ratten V. Athletes as entrepreneurs: the role of social capital and leadership ability. Int J Entrep Small Bus. (2015) 25(4):442–59. 10.1504/IJESB.2015.070217

[21] Pellegrini MM Rialti R Marzi G Caputo A. Sport entrepreneurship: a synthesis of existing literature and future perspectives. Int Entrep Manag J. (2020) 16(3):795–826. 10.1007/s11365-020-00650-5

[22] Ratten V. Sport Entrepreneurship: Developing and Sustaining an Entrepreneurial Sports Culture. Cham: Springer (2018).

[23] Hoye R Smith A Nicholson M Stewart B. Sport Management: Principles and Applications. 5th ed. Abingdon: Routledge (2018).

[24] Eric Boyd D Keith Harrison C McInerny H. Transitioning from athlete to entrepreneur: an entrepreneurial identity perspective. J Bus Res. (2021) 136:479–87. 10.1016/j.jbusres.2021.07.010

[25] Geeraert A. National sports governance observer 2018. Play the Game/Danish Institute for Sports Studies. (2018).

[26] Creswell JW Poth CN. Qualitative Inquiry and Research Design: Choosing among Five Approaches. 4th ed. Thousand Oaks, CA: Sage Publications (2018).

[27] Tomás J Sousa H Araújo D Field A Ribeiro J Sarmento H. Crossing the line: players’ perspectives on the leap from junior to senior football. Int J Sports Sci Coach. (2026). 10.1177/17479541261441668

[28] Schwandt TA. Constructivist, interpretivist approaches to human inquiry. In: Denzin NK Lincoln YS, editors. Handbook of Qualitative Research. Thousand Oaks, CA: Sage (1994). p. 118–37.

[29] Denzin NK Lincoln YS. The Sage Handbook of Qualitative Research. 5th ed. Thousand Oaks, CA: Sage Publications (2018).

[30] Thomas R Lynn P. Survey Research in Practice. London: SAGE (2020).

[31] Lincoln YS Guba EG. Naturalistic Inquiry. Beverly Hills, CA: Sage (1985).

[32] Halcomb EJ Davidson PM. Is verbatim transcription of interview data always necessary? Appl Nurs Res. (2006) 19(1):38–42. 10.1016/j.apnr.2005.06.001 16455440

[33] Kallio H Pietilä AM Johnson M Kangasniemi M. Systematic methodological review: developing a framework for a qualitative semi-structured interview guide. J Adv Nurs. (2016) 72(12):2954–65. 10.1111/jan.13031 27221824

[34] Braun V Clarke V. Using thematic analysis in psychology. Qual Res Psychol. (2006) 3(2):77–101. 10.1191/1478088706qp063oa

[35] Baron RA Franklin RJ Hmieleski KM. Why entrepreneurs often experience *low*, not high, levels of stress: the joint effects of selection and psychological capital. J Manage. (2016) 42(3):742–68. 10.1177/0149206313495411

[36] Brown DJ Glastetter-Fender C Shelton M. Career development of student athletes: a conceptual model. J Career Dev. (2020) 47(3):305–18. 10.1177/0894845318793232

[37] Cosh S Crabb S Tully PJ. Athlete identity and mental health following retirement from sport: a systematic review. Int Rev Sport Exerc Psychol. (2021) 14(1):1–27. 10.1080/1750984X.2020.1851242

[38] Haslam SA Jetten J Cruwys T Dingle G Haslam C. The new Psychology of Health: Unlocking the Social Cure. Abingdon: Routledge (2020).

[39] Torregrosa M Ramis Y Pallarés S Azócar F Selva C. Olympic Athletes back to retirement: a qualitative longitudinal study. Psychol Sport Exerc. (2020) 47:101618. 10.1016/j.psychsport.2019.101618

[40] Fleischman D Mulcahy R English P Kirby K Stevenson T Sotiriadou P. Athlete career transitions: a systematic review and future directions. Eur Sport Manag Q. (2025) 25(5):768–92. 10.1080/16184742.2024.2437793

[41] United States Olympic and Paralympic Committee. Professional Development Programs. Colorado Springs, CO: United States Olympic and Paralympic Committee. (Ajouter l'URL officielle si ce document a été consulté en ligne.) (2024).

[42] Arrieta J. Les sportifs de haut niveau face au double projet: Entre volontés d’accompagnement, contraintes organisationnelles et innovations. Le cas d’Université Côte d’Azur [Mémoire de master, Université Côte d’Azur]. (2022).

[43] Borrueco M Jordana A Ramis Y Regüela S de Brandt K Vitali F. A European Need Analysis on Athletes’ employment After Sports Retirement (EP-NEST Report). European Parliament (2024).

[44] Choi J Kim H-D. Sustainable careers of athletes: themes and concepts regarding transition theories involving athletes. Sustainability. (2021) 13(9):4824. 10.3390/su13094824

[45] National Collegiate Athletic Association. Educational and Leadership Opportunities. NCAA (2024).

[46] National Collegiate Athletic Association. GOALS study: Career Planning and Development Report. NCAA (2026).

[47] Lefebvre du Grosriez S Cécé V Isoard-Gautheur S Martinent G Sarrazin P. Measuring sport-school conflict and enrichment: theoretical considerations and new measures. OSF Preprints. (2022, décembre). 10.31219/osf.io/rch7g

[48] Wylleman P. A developmental and holistic perspective on transitioning out of elite sport. In: M. H. Anshel (Ed.), APA handbook of sport and exercise psychology: Vol. 1. Sport psychology. American Psychological Association (2019). pp. 201–16.

[49] Küttel A Larsen CH. Risk and protective factors for mental health in elite athletes: a scoping review. Int Rev Sport Exerc Psychol. (2020) 13(1):231–65. 10.1080/1750984X.2019.1689574

[50] Ryan RM Deci EL. Self-determination theory: basic psychological needs in motivation, development, and wellness (2e éd.). Guilford Press (2023).

